# Endovascular treatment versus standard medical treatment in patients with established large infarct: a cohort study

**DOI:** 10.1097/JS9.0000000000001539

**Published:** 2024-05-08

**Authors:** Changwei Guo, Linyu Li, Jiandi Huang, Jie Yang, Jiaxing Song, Jiacheng Huang, Zhouzhou Peng, Nizhen Yu, Chang Liu, Weilin Kong, Jinrong Hu, Li Chen, Meng Guo, Chengsong Yue, Dahong Yang, Xiang Liu, Jian Miao, Mengmeng Wang, Xiangyun Luo, Zhaoyin Tang, Xiubing Bai, Duolao Wang, Fengli Li, Qingwu Yang, Wenjie Zi

**Affiliations:** a Department of Neurology, Xinqiao Hospital and the Second Affiliated Hospital of Army Medical University; b Department of Neurology, The Second Affiliated Hospital of Chongqing Medical University, Chongqing; c Department of Neurology, Xianyang Hospital of Yan’an University, Xianyang; d Department of Neurology, Weihai Municipal Hospital, Cheeloo College of Medicine, Shandong University, Shandong; e Affiliated Hospital of Weifang Medical University, School of Clinical Medicine, Weifang Medical University, Weifang, People’s Republic of China; f Global Health Trials Unit, Liverpool School of Tropical Medicine, Liverpool, UK

**Keywords:** acute ischemic stroke, endovascular treatment, large core infarction

## Abstract

**Background:**

Previous trials confirmed the benefit of endovascular treatment (EVT) in acute large core stroke, but the effect of EVT on outcomes in these patients based on noncontrast computed tomography (NCCT) in real-world clinical practice was unclear. The aim of this study was to explore the effect of EVT versus standard medical treatment (SMT) in patients with large ischemic core stroke defined as Alberta Stroke Program Early CT Score (ASPECTS) ≤5 based on NCCT alone.

**Materials and methods:**

Patients with acute large core stroke at 38 Chinese centers between November 2021 and February 2023 were reviewed from a prospectively maintained database. The primary outcome was favorable functional outcome [modified Rankin Scale score (mRS), 0–3] at 90 days. Safety outcomes included 48 h symptomatic intracerebral hemorrhage (sICH) and 90-day mortality.

**Results:**

Of 745 eligible patients recruited at 38 stroke centers between November 2021 and February 2023, 490 were treated with EVT+SMT and 255 with SMT alone. One hundred and eighty-one (36.9%) in the EVT group achieved favorable functional independence versus 48 (18.8%) treated with SMT only [adjusted risk ratio (RR), 1.86; 95% CI: 1.43–2.42, *P*<0.001; adjusted risk difference (RD), 13.77; 95% CI: 7.40–20.15, *P*<0.001]. The proportion of sICH was significantly higher in patients undergoing EVT (13.3 vs. 2.4%; adjusted RR, 5.17; 95% CI: 2.17–12.32, *P*<0.001; adjusted RD, 10.10; 95% CI: 6.12–14.09, *P*<0.001). No significant difference of mortality between the groups was observed (41.8 vs. 49.0%; adjusted RR, 0.91; 95% CI: 0.77–1.07, *P*=0.24; adjusted RD, −5.91; 95% CI: −12.91–1.09, *P*=0.1).

**Conclusion:**

Among patients with acute large core stroke based on NCCT in real-world, EVT is associated with better functional outcomes at 90 days despite of higher risk of sICH. Rates of procedure-related complications were relatively higher in the EVT+SMT group.

## Introduction

Highlights

- Our analysis found that the use of endovascular treatment resulted in better functional outcomes at 90 days despite of higher risk of symptomatic intracranial hemorrhage and complications for patients with large infarctions defined as Alberta Stroke Program Early Computed Tomography Score (ASPECTS) of 0 to 5 based on noncontrast CT selection.
- Several landmark studies have demonstrated the efficacy of endovascular treatment for patients with large infarction for selected. Even so, a low rate of modified Rankin Scale (mRS) of 0–3 (31–47%) and an uncertain range of symptomatic intracerebral hemorrhage (0.6–9%) make it easy to raise a rational fear that how much of the effectiveness of randomized controlled trials confirming EVT in patients with large infarction translates into the benefit of patients in real-world medical practice.
- The data that support the findings of this study are available from the corresponding author upon reasonable request.

Acute ischemic stroke with large cores accounts for ~20% of large vessel occlusion strokes but usually causes catastrophic medical condition, such as bedridden, incontinent, or even death^1^. Patients with large ischemic cores, defined by the Alberta Stroke Program Early Computed Tomography Score (ASPECTS) of 0–5 or ischemic core ≥50 ml, are ineligible for endovascular treatment (EVT) according to current American and European guidelines due to wide early ischemic injury and less possibility to achieve functional independence^2–4^.

Recently, four landmark stroke trials, Endovascular Salvage for Cerebral Ultra-acute Embolism—Japan Large Ischemic Core Trial (RESCUE-Japan LIMIT)^5^, randomized controlled trial to optimize patient’s selection for endovascular treatment in acute ischemic stroke (SELECT 2)^6^, endovascular therapy in acute anterior circulation large vessel occlusive patients with a large infarct core (ANGEL-ASPECT)^7^, and endovascular thrombectomy for acute ischemic stroke with established large infarct (TENSION)^8^ have confirmed the safety and efficacy of EVT combined with standard medical treatment (SMT) in patients with large ischemic burden compared with SMT-alone. The intention-to-treat population analysis of the primary outcome in the Thrombectomy for Emergent Salvage of Large Anterior Circulation Ischemic Stroke (TESLA) failed to demonstrated efficacy of EVT in patients with a large-core infarction on the basis of ASPECTS 2–5 according to noncontrast computer tomography (NCCT), but the results of secondary outcome including the proportion of mRS score of 0–3 at 90 days and rate of major neurological improvement highlighted a strong suggestion in favor of EVT^9^. Even so, a low rate of modified Rankin Scale (mRS) of 0 to 3 (31–47%) and an uncertain range of symptomatic intracerebral hemorrhage (0.6–9%) make it easy to raise a rational fear that how much of the effectiveness of randomized controlled trials confirming EVT in patients with large infarction translates into benefit of patients in real-world medical practice^1,10,11^.

The enrolled patients of previous trials were strictly screened mainly by advanced imaging with MRI or computed tomography perfusion (CTP). Advanced imaging could identify patients with large core but wide penumbra that could be salvaged through EVT^11,12^. But strict advanced imaging selection may exclude the patients that could benefit from EVT and even make delay in treatment to increase the chance of futility^13^. Besides, access to urgent MRI or CTP is not universally available in many stroke centers, especially in developing countries^14^. Conversely, NCCT is more available at stroke centers in clinical practice. Previous studies did not observed significant differences in the clinical outcomes of patients selected with NCCT compared with those selected with advanced imaging^15,16^. Therefore, the present study aimed to explore the association between EVT combined with SMT and clinical outcomes in patients with large cores according to NCCT compared to SMT-alone in real-world.

## Material and methods

### Study cohort and patients

This study was a subanalysis of a prospective multicenter corhort study and patients treated between 1 November 2021 and 8 February 2023. The registry was an ongoing, prospective, observational, nationwide registry including all patients with acute large vessel occlusion within 24 h from the point that they were last known well and undergoing standard treatment in China (registered at the https://www.chictr.org.cn/). The study protocol was approved by ethics committee of the leading center and the local committees of the participating hospitals gave approval as well. All patients or their legally authorized representatives provided signed, informed consent.

The inclusion criteria for this analysis were as follows: (1) an age at least 18 years old; (2) acute ischemic stroke due to anterior circulation large vessel occlusion, defined as occlusion of the internal carotid artery (ICA) or the M1 segment or M2 segment of the middle cerebral artery; (3) large ischemic core on NCCT (defined as an ASPECTS of 0–5); (4) within 24 h of stroke onset or last known within 24 h (the time metric of time last known well within 24 h was used instead if the presentation time was unavailable). Patients were excluded from the study in the case of (1) prestroke mRS >2; (2) lack of follow-up information on 90-day outcomes; (3) serious or terminal illness that was not related to acute ischemic stroke.

### Treatments

Patients were divided into the SMT-alone and EVT plus SMT group. The SMT-alone group received SMT including intravenous thrombolysis (IVT, the dose of alteplase was 0.9 mg/kg for Alteplase and 0.25 mg/kg for Tenecteplase), antiplatelet drugs, anticoagulation drugs, or combination of these treatments according to the guidelines for the management of acute ischemic stroke^17^. EVT included stent retrievers, aspiration, balloon angioplasty, stenting, intraarterial thrombolysis, mechanical fragmentation, or any combinations of these approaches. The decision to perform EVT+SMT or SMT alone was left to the discretion of the local physicians. Decisions to perform decompressive hemicraniectomy in patients with severe brain swelling were made in accordance with local practices.

### Data collection

Patients’ baseline demographic characteristics, stroke risk factors, laboratory findings, stroke severity [based on the National Institutes of Health Stroke Scale (NIHSS)^18^], collateral status [based on the American Society of Interventional and Therapeutic Neuroradiology/Society of Interventional Radiology collateral grading system (ASITN/SIR)^19^], time from symptom onset or last known well to imaging, groin puncture and recanalization, EVT technique, complications, reperfusion grades, presumed stroke causative mechanism [based on the Trial of ORG10172 in Acute Stroke Treatment (TOAST) classification^20^], location of occlusion, and baseline core infarct determined by the NCCT-based ASPECTS were recorded.

### Imaging assessment

The imaging core laboratory evaluated the findings on baseline NCCT for the ASPECTS, baseline imaging (computed tomographic angiography, magnetic resonance angiography, or digital subtraction angiography) for the occlusion site, angiographic outcomes on digital subtraction angiography imaging for technical efficacy outcomes regarding reperfusion, and the follow-up computed tomography within 48 h for the presence of intracranial hemorrhage. Successful reperfusion was defined as the restoration of blood flow to greater than 50% (2b to 3) of the involved territory, as assessed with the use of the modified Treatment in Cerebral Ischemia classification [mTICI, scores range from 0 (no flow) to 3 (normal flow)^21^]. Baseline imaging, reperfusion grades, and postprocedural imaging were independently evaluated by the imaging core laboratory who were blind to the treatment groups and clinical outcomes.

### Clinical outcomes

The primary outcome was favorable functional outcome, defined as a mRS of 0–3 at 90 days, which was recorded during a follow-up visit or telephone encounter at 90 days after stroke by local physicians or registered nurse. Secondary outcomes included ordinal score on mRS at 90 days, functional independence (defined as mRS of 0–2), the proportion of mRS 0–4, successful reperfusion. Safety outcomes included the incidence of death within 90 days, symptomatic intracranial hemorrhage (SICH) within 48 h according to the Heidelberg Bleeding Classification^22^, and any intracranial hemorrhage within 48 h.

### Statistical analysis

Baseline characteristics, treatment profiles, time metrics were reported according to the treatment arms. Data were presented as medians [interquartile ranges (IQRs)] or numbers with percentages, unless otherwise indicated. Univariate analysis was performed using the Kruskal–Wallis test, *χ* ^2^ test, or Fisher exact test, as appropriate. Missing baseline covariates were imputed using the hot deck methods in the covariate adjusted analysis based on the covariate distributions. Only a small number of patients needed the hot deck imputation; therefore, the techniques recommended in (24) for a variance estimate that incorporates the additional variance from the missing information was not implemented.

For efficacy and safety outcomes assessment between patients treated with EVT+SMT and those with SMT-alone, propensity score matching (PSM) methods were used to balance prognostic important factors. The propensity score was estimated using a multivariable logistic regression model, with the treatment received as the dependent variable and age, history of hypertension, hyperlipidemia, diabetes, baseline ASPECTS, baseline NIHSS, systolic blood pressure, diastolic blood pressure, intravenous thrombolysis, ASITN/SIR, stroke mechanism, occlusion sites, time from last known well to imaging as covariates. We performed a 1:1 matching based on the nearest neighbor matching with a 0.2 caliper.

The multivariable models were adjusted for age, history of hypertension, hyperlipidemia, diabetes, baseline ASPECTS, baseline NIHSS, intravenous thrombolysis, ASITN/SIR, stroke mechanism, occlusion sites, time from last known well to imaging, systolic blood pressure, diastolic blood pressure. The generalized linear models were used as the primary analysis. Models with robust error estimators with the Poisson distribution and log link function were used to estimate the risk ratio (RR), and with the Gaussian distribution and identity link function were used to estimate the risk difference (RD). For the comparison of the distributions of the mRS scores at 90 days, ordered logistical regression was used to estimate the common odds ratio. Besides, two assumption-free method, the Wilcoxon–Mann–Whitney generalized odds ratio and win ratio approaches was used for the comparison of the distribution of the mRS scores for sensitivity analysis^23,24^. Besides, generalized linear mixed models were used take into account of center effect and pair effect in sensitivity. Generalized estimating equation were also used as sensitivity analysis to account center-effect.

In the inverse probability of treatment weighting (IPTW) cohort, the treatment effect was estimated with the inversed probability-weighted regression adjustment model, which used the inversed propensity score to weight each subject, and adjusted for the weighted regression coefficients to compute the averages of treatment-level predicted outcomes. Using the doubly robust estimation to reduce the bias and be less sensitive to misspecification^25^. The primary analysis of the primary outcome were based on the IPTW analysis.

We further investigated the heterogeneity in treatment effect size for the primary outcome within the following subgroups: age (≤75 vs. >75 years old), sex (female vs. male), baseline NIHSS score (≤17 vs. >17), ASPECTS (≤2 vs. >2), IVT (no vs. yes), occlusion site, stroke causative mechanism, time from last known well to imaging (≤360 vs. >360 min). A multiplicative term was entered into regression models to estimate the significance of the interaction with the treatment assignment.

In addition, an instrumental variable analysis (IVA) was performed to evaluate the association of treatment allocation with clinical outcomes. The center-level preference for EVT, which is defined as the proportion of EVT for all patients at a particular center, was used as the instrument. A two-stage residual inclusion approach was employed: in the first stage, an expectation of treatment allocation based on co-variables and instrumental variable was estimated, and the co-variables were the same as in the other adjusted model; then, in the second stage, outcomes were predicted based on original treatment allocation, covariates, and residuals from the first-stage regression.

All statistical tests were two-sided, with *P*-values <0.05 considered statistically significant. Statistical analyses were conducted in SAS 9.4 and STATA 17. All the work has been reported in line with the strengthening the reporting of cohort, cross-sectional, and case–control studies in surgery (STROCSS) criteria^26^ (Supplemental Digital Content 1, http://links.lww.com/JS9/C498).

## Results

### Patients cohort and baseline characteristics

**Figure 1:**
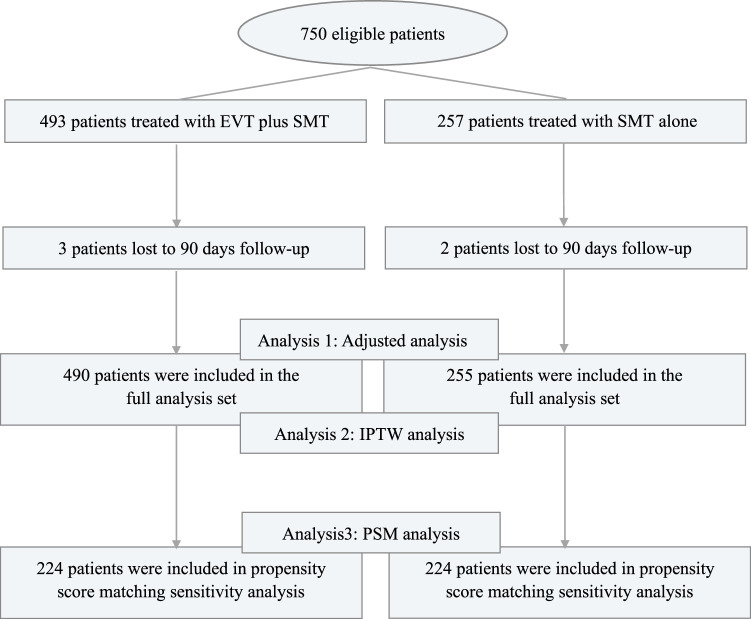
Flowchart.

Totally, 745 eligible patients were eligible and consented from the prospective study between November 2021 and February 2023, from 38 stroke centers across China. A total of 255 patients received SMT alone, while 490 treated with EVT plus SMT. Figure 1 shows a flowchart of patients enrolled in this study. (Power were analyzed in Figure S1, Supplemental Digital Content 2, http://links.lww.com/JS9/C499).

Table 1 shows baseline characteristics of the eligible patients. Overall, age was median 70 [interquartile (IQR) 61–78] years, baseline NIHSS 17 (IQR 14–21) and ASPECTS 4 (IQR 2–5). Compared with the SMT-alone group, patients in the EVT group had a younger age [69 (59–78) years vs. 72 (65–79) years; *P*<0.001], lower proportion of hypertension [181 of 255 (71.0%) vs. 297 of 490 (60.6%); *P*=0.005], higher proportion of hyperlipidemia [38 of 255 (14.9%) vs. 106 of 490 (21.6%); *P*=0.03], higher ASPECTS score [3 (1–5) vs. 4 (2–5); *P*<0.001], poorer collateral status [ASITIN/SIR: 2 (1–3) vs. 2 (1–2); *P*=0.02], lower systolic blood pressure levels [155 (136–178) vs. 146 (128–164); *P*<0.001], lower diastolic blood pressure levels [88 (79–101) vs. 86 (75–96); *P*=0.006], and a significant difference of presumed stroke mechanism [e.g. cardioembolism: 109 of 255 patients (42.7%) vs. 277 of 490 patients (56.5%); *P*<0.001] and occlusion sites [ICA: 66 of 255 (25.9%) vs. 206 of 490 (42.0%); M1: 159 of 255 (62.4%) vs. 233 of 490 (47.6%); M2: 30 of 255 (11.8%) vs. 51 of 490 (10.4%); *P*<0.001]. Other baseline characteristics were not statistically different between the two groups.

**Table 1:** Baseline characteristics of the patients.

Characteristics	All (*n*=745)	EVT+SMT (*n*=490)	SMT (*n*=255)	*P*
Age, median (IQR), years	70 (61–78)	69 (59–78)	72 (65–79)	<0.001
Sex — no. (%)				0.18
Male	414 (55.6)	281 (57.3)	133 (52.2)	
Female	331 (44.4)	209 (42.7)	122 (47.8)	
Medical History — no. (%)				
Atrial fibrillation	329 (44.2)	221 (45.1)	108 (42.4)	0.47
Hypertension	478 (64.2)	297 (60.6)	181 (71.0)	0.005
Hyperlipidemia	144 (19.3)	106 (21.6)	38 (14.9)	0.03
Diabetes	125 (16.8)	73 (14.9)	52 (20.4)	0.06
Smoking	222 (29.8)	151 (30.8)	71 (27.8)	0.40
Blood pressure on admission, median (IQR), mmHg^a^				
Systolic	149 (131–168)	146 (128–164)	155 (136–178)	<0.001
Diastolic	86 (77–98)	86 (75–96)	88 (79–101)	0.006
Glucose, median (IQR), mmol/l ^b^	7.1 (6.0–8.8)	7.2 (5.9–8.9)	7.1 (6.0–8.6)	0.67
Baseline NIHSS score, median (IQR)	17 (14–21)	17 (14–20)	17 (13–22)	0.84
Baseline ASPECTS, median (IQR)	4 (2–5)	4 (2–5)	3 (1–5)	<0.001
0–2	246 (33.0)	135 (27.6)	111 (43.5)	
3–5	499 (67.0)	355 (72.4)	144 (56.5)	
Left hemisphere affected — no. (%)	365 (49.0)	248 (50.6)	117 (45.9)	0.22
Intravenous thrombolysis — no. (%)	201 (27.0)	122 (24.9)	79 (31.0)	0.08
ASTIN/SIR grade ^c^, median (IQR)	2.0 (1.0, 2.0)	2.0 (1.0, 2.0)	2.0 (1.0, 3.0)	0.02
0–1	343 (46.2)	239 (48.8)	104 (41.1)	
2	247 (33.2)	169 (34.5)	78 (30.8)	
3–4	153 (20.6)	82 (16.7)	71 (28.1)	
Stroke causative mechanism — no. (%)				<0.001
Large artery atherosclerosis	269 (36.1)	146 (29.8)	123 (48.2)	
Cardioembolism	386 (51.8)	277 (56.5)	109 (42.7)	
Other	25 (3.4)	20 (4.1)	5 (2.0)	
Unknown	65 (8.7)	47 (9.6)	18 (7.1)	
Occlusion location — no. (%)				<0.001
Internal carotid artery	272 (36.5)	206 (42.0)	66 (25.9)	
M1 segment	392 (52.6)	233 (47.6)	159 (62.4)	
M2 segment	81 (10.9)	51 (10.4)	30 (11.8)	
Tandem occlusions — no. (%)	53 (7.1)	36 (7.3)	17 (6.7)	0.73
General anesthesia — no. (%)	—	85 (17.3)	—	
Last seen well to imaging time, median (IQR), min ^d^	302.5 (161–499)	292.5 (158–458)	307.5 (165.5–526.5)	0.14
Last seen well to puncture time, median (IQR), min ^e^	—	362 (240–542)	—	
Last seen well to recanalization time, median (IQR), min ^f^	—	449.5 (326–654.5)	—	

^a^ Data on blood pressure on admission were missing for eight patients in the EVT group.

^b^ Data on glucose were missing for 12 patients in EVT group and eight patients in SMT group.

^c^ Data on ASTIN/SIR grade were missing for two patients in the SMT group.

^d^ Data on last seen well to imaging time were missing for seven patients in the SMT group.

^e^ Data on last seen well to puncture time were missing for five patients in the EVT group.

^f^ Data on last seen well to recanalization time were missing for six patients in the EVT group.

After PSM, baseline characteristics between the groups were generally balanced. Details are available in Supplementary Table S1, (Supplemental Digital Content 2, http://links.lww.com/JS9/C499) and Supplementary Figure S2 (Supplemental Digital Content 2, http://links.lww.com/JS9/C499). A total of 224 patients who had EVT plus SMT were evaluable for the matched-pairs analysis with the multivariable method.

### Primary efficacy outcome

**Figure 2:**
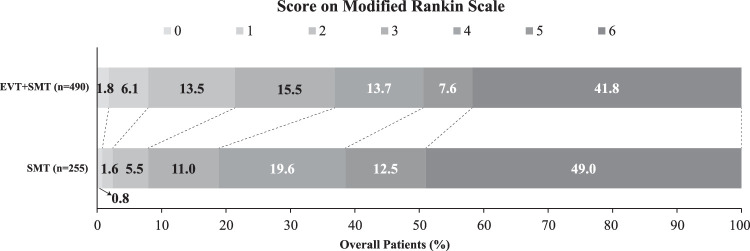
Distribution of the Modified Rankin Scale score at 90 days.

EVT plus SMT was associated with favorable functional outcome at 90 days in 36.9% (181 of 490) patients in the EVT plus SMT group and 18.8% (48 of 255) in the SMT group (adjusted RR, 1.86; 95% CI: 1.43–2.42; *P*<0.001; adjusted RD, 13.77; 95% CI: 7.40–20.15, *P*<0.001; Table 2 and Fig. 2). In the primary analysis using the IPTW cohort (Figure S3, Supplemental Digital Content 2, http://links.lww.com/JS9/C499), primary outcome was consistent with original primary analysis after PSM (Supplementary Figure S4, Supplemental Digital Content 2, http://links.lww.com/JS9/C499), compared with SMT-alone group, the proportion of favorable functional outcome at 90 days in the EVT plus SMT group was significantly higher (Table 2, Supplementary Table S2, Supplemental Digital Content 2, http://links.lww.com/JS9/C499) (Table 2).

**Table 2:** Primary and secondary efficacy outcomes.

	Before matching						IPTW		PSM	
Outcomes	All	EVT+SMT	SMT	Treatment effect	Effect value	*P*	Effect value	*P*	Effect value	*P*
Primary outcome										
Modified Rankin scale score of 0–3 at 90 d — no./total no. (%)	229 (30.7)	181 (36.9)	48 (18.8)	Risk ratio	1.86 (1.43–2.42)	<0.001	1.96 (1.48–2.60)	<0.001	1.79 (1.35–2.37)	<0.001
				Risk Difference	13.77 (7.40–20.15)	<0.001	15.20 (8.69– 21.71)	<0.001	13.64 (6.36–20.93)	<0.001
Secondary outcome										
Score on the modified Rankin scale at 90 days (IQR)	5 (3–6)	4 (3–6)	5 (4–6)	Common odds ratio	1.79 (1.30–2.50)	<0.001	2.10 (1.71–2.59)	<0.001	1.74 (1.20–2.51)	0.004
				Generalized odds ratio	1.40 (1.19–1.64)	<0.001	—	—	1.29 (1.06–1.59)	0.01
				Win ratio	1.59 (1.28–2.00)	<0.001	—	—	1.43 (1.08–1.92)	0.01
Modified Rankin scale score of 0–2 at 90 days — no./total no. (%)	229 (30.7)	181 (36.9)	48 (18.8)	Risk ratio	2.47 (1.61–3.81)	<0.001	2.85 (1.86–4.39)	<0.001	1.90 (1.16–3.13)	0.01
				Risk difference	10.33 (5.32–15.35)	<0.001	11.49 (6.44–16.54)	<0.01	5.86 (0.23–11.49)	0.04
Modified Rankin scale score of 0–4 at 90 days — no./total no. (%)	346 (46.4)	248 (50.6)	98 (38.4)	Risk ratio	1.28 (1.09–1.52)	0.003	1.39 (1.15–1.66）	<0.001	1.25 (1.03–1.50)	0.02
				Risk difference	8.74 (1.94–15.55)	0.01	11.34 (4.34–18.34)	<0.001	7.93 (0.17–15.69)	0.045
Successful reperfusion	—	423 (86.3)	—							
Safety outcome										
Symptomatic intracranial hemorrhage within 48 h— no./total no. (%)	71 (9.5)	65 (13.3)	6 (2.4)	Risk ratio	5.17 (2.17–12.32)	<0.001	3.56 (1.29–9.78)	0.01	4.33 (1.78–10.55)	<0.001
				Risk Difference	10.10 (6.12–14.09)	<0.001	8.99 (4.39–13.59)	<0.001	8.61 (4.00–13.23)	<0.001
Death within 90 days — no./total no. (%)	330 (44.3)	205 (41.8)	125 (49.0)	Risk ratio	0.91 (0.77–1.07)	0.24	0.84 (0.72–0.99)	0.036	0.92 (0.76–1.10)	0.35
				Risk Difference	−5.91(−12.91–1.09)	0.10	−9.32 (−16.53–−2.13）	0.01	−5.33 (−13.29–2.62)	0.19
Any intracranial hemorrhage within 48 hours — no./total no. (%)	208 (27.9)	180 (36.7)	28 (11)	Risk ratio	3.43 (2.36–4.99)	<0.001	3.40 (2.12–5.45)	<0.001	3.37 (2.26–5.02)	<0.001
				Risk difference	26.17 (19.94–32.41)	<0.001	25.11 (18.72–31.49)	0.001	25.66 (18.29–33.02)	<0.001

### Secondary efficacy outcomes

Secondary clinical efficacy outcomes are shown in Table 2. There was a shift toward better outcomes (lower mRS scores) across the mRS categories with EVT plus SMT (common OR, 1.79; 95% CI: 1.30–2.50; *P*<0.001; generalized OR, 1.40, 95% CI: 1.19–1.64, *P*<0.001; win ratio, 1.59, 95% CI: 1.28–2.00, *P*<0.001; Table 2 and Fig. 2). EVT plus SMT was associated with independent functional outcome at 90 days [20 of 225 (7.8%) vs. 105 of 490 (21.4%); adjusted RR, 2.47; 95% CI: 1.61–3.81; *P*<0.001; adjusted RD, 10.33; 95% CI: 5.32–15.35, *P*<0.001). Two hundred five of 490 patients (50.6%) in the EVT plus SMT group achieved a 90-day mRS of 0 to 4 and 98 of 255 patients in the SMT group had a mRS of 0 to 4 at 90 days (adjusted RR, 1.28; 95% CI: 1.09–1.52; *P*=0.003; adjusted RD, 8.74; 95% CI: 1.94–15.55, *P*=0.01). The treatment effect remain robust in the PSM and IPTW analysis.

### Safety outcomes

There was a numerically lower but not significantly different rate of 90-day-mortality with EVT plus SMT [125 of 255 (49.0%) vs. 205 of 490 (41.8%); adjusted RR, 0.91; 95% CI: 0.77–1.07; *P*=0.24; adjusted RD, −5.91, 95% CI: −12.91–1.09, *P*=0.10). The rate of SICH was 13.3% (65 of 490 patients) in the EVT plus SMT group and 2.4% (6 of 255 patients) in the SMT-alone group (adjusted RR, 5.17, 95% CI: 2.17–12.32, *P*<0.001; RD, 10.10, 95% CI: 6.12–14.09, *P*<0.001). Rates of any intracranial hemorrhage, herniation, and craniectomy were significantly higher in the EVT plus SMT group compared with SMT-alone group (Supplementary Table S3 in the Supplement, Supplemental Digital Content 2, http://links.lww.com/JS9/C499). Similar safety outcomes were observed after PSM and in the IPTW cohort.

### Sensitivity analysis

Using the IVA model in sensitivity analysis (Supplementary Table S4 in the Supplement, Supplemental Digital Content 2, http://links.lww.com/JS9/C499), the Wald F-statistic for center proportion of EVT plus SMT was 217.51, suggesting a strong instrument. There was a significant association between EVT plus SMT and independent ambulation at 90 days. In addition, EVT plus SMT was associated with all the secondary efficacy outcomes. There was no significant difference in mortality between the two groups, the rates of SICH and any intracranial hemorrhage were significantly higher in the EVT plus SMT groups. Consistent outcomes were observed in the generalized estimating equation analysis and generalized linear mixed effect model (Supplementary Table S5, Supplemental Digital Content 2, http://links.lww.com/JS9/C499 and Supplementary Table S6 in the Supplement, Supplemental Digital Content 2, http://links.lww.com/JS9/C499).

### Subgroup analysis

**Figure 3:**
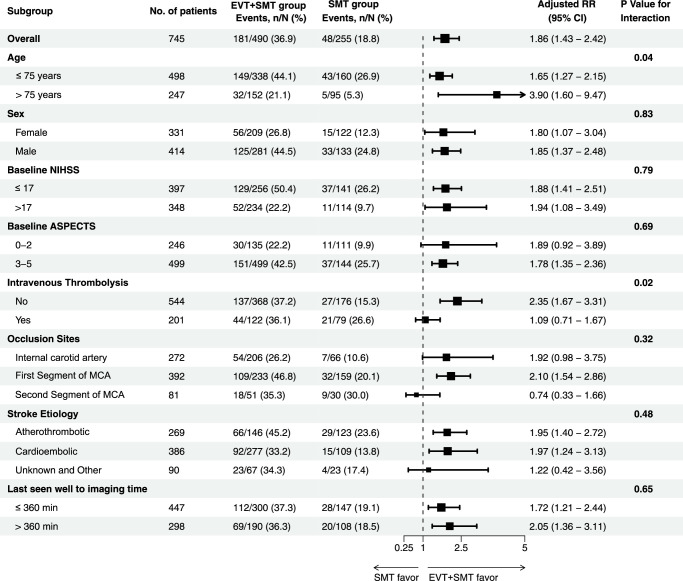
Subgroup analysis.

Subgroup analyses were based on the full data set. The relation between the occurrence of the favorable functional outcome at 90 days and EVT plus SMT was consistent across subgroups. Potential treatment heterogeneity was found in age and IVT. For example, in patents with an age of more than 75 years, EVT plus SMT were associated with higher treatment effect (adjusted RR 3.90, 95% CI: 1.60–9.47) than in patients with an age of no more than 75 years (adjusted RR 1.65, 95% CI: 1.27–2.15) (Fig. 3). No statistical heterogeneity was found in patients with different sex, different baseline ASPECTS, baseline NIHSS, occlusion sites, stroke etiology, and last seen well to imaging time. Moreover, we have conducted additional analysis for the outcomes of each EVT tech in the Supplement (Supplementary Table S7-11, Supplemental Digital Content 2, http://links.lww.com/JS9/C499).

## Discussion

Our results suggest that, in the real-word practice, EVT may improve clinical functional outcomes in patients with large vessel occlusion presenting with large ischemic core (ASPECTS ≤5 on NCCT) within 24 h of symptom onset or last known well despite of high risk of symptomatic intracerebral hemorrhage.

Several observational studies have investigated the effect of imaging modality (NCCT vs. CTP/MRI) on the selection of EVT in AIS patients^15,16,27–29^. However, the results were inconsistent, with some indicating similar functional outcomes between the two imaging modalities^15,16,27–29^, while others showed improved outcomes in patients selected by advanced imaging paradigms. Moreover, these studies were based on patients with mild or moderate infarction, whether the result can be extended to patients with large core infarction remain unclear. Previous trials of EVT in patients with large core stroke mainly based on advanced imaging^5–7^. However, none of the participants of the previous studies were enrolled based on NCCT alone. Nearly 86% of patients included in the RESCUE-Japan LIMIT with an ASPECTS value were based on MRI, which have been showed to be more sensitive to identify ischemic regions but overestimating ischemic core volumes compared with NCCT. Moreover, MRI-based ASPECTS was reported 1 scale lower than that measured by NCCT^30^. Most of the enrolled patients in the ANGLE-ASPECT trial were screened by CTP. Advanced imaging selection is beneficial to improve clinical outcomes of patients with large core, but this selection may make delay in treatment and deny many patients who could benefit from EVT. In these trials, nearly only three patients of 10 large core patients with EVT are functional independent, as NCCT is available at all stroke centers, how about the effect of EVT on clinical outcomes in patients with large core evaluated by NCCT alone? In the EVT group of our studies, favorable outcome occurred in 36.9% of the patients. This result was slightly lower than that of the SELECT 2 trial, which mostly used more generalizable imaging triage methods (NCCT). This can be explained that our study enrolled patients with ASPECTS 0–5, but only patients with ASPECTS 3–5 were enrolled in the SELECT trial, as low ASPECTS rating on NCCT predicts poor outcome after reperfusion^31^. In a secondary analysis of the RESCUE-Japan LIMIT, EVT was not associated with improved clinical outcomes at 90 days in patients with large core stroke and ASPECTS 3 or less^32^.

Although, EVT is associated with improved clinical outcomes in our study, death occurred in more than 40% of patients despite of EVT, and there is no significant difference between the two groups. It still remains a great challenge for both relatives of patients and neurointerventionists to decide whether to perform EVT considering a high chance of death and high cost. In the RESCUE-Japan LIMIT and ANGLE-ASPECT trial, ~20% of death within 90 days were reported, which was much less than that of our study. This could be explained that advance imaging selection excluded those patients with more opportunity to achieve poor outcome or even death. However, mRS of 5 occurred in 37 (7.6%) patients in the EVT group and 32 (12.5%) patients in the SMT group in our study, which suggests that EVT may decrease the opportunity of outcome of bedridden and incontinent. To some degree, EVT may improve the quality of lives among the survivors.

However, the EVT was associated higher risk of complications such as sICH. In our study, the rate of SICH was 13.3% in the EVT group, which was significantly higher than the SMT group. Previous study reported 11.2% of SICH in patients with ASPECTS 2 to 5 after EVT^10^. In the recent clinical trials, SICH occurred in 0.6–9% patients treated with EVT, which is much less than that of our study^5–7^. This could be explained as followed. First, patients with low ASPECTS are at higher risk of SICH^33^. In our study, 27.6% of patients in the EVT group presented with ASPECTS 0 to 2. All of the previous trials excluded those patients with low ASPECTS (0–2) due to high risk of SICH. A prespecified secondary analysis of the RESCUE-Japan LIMIT trial showed that SICH occurred in 10.7% patients among those with ASPECTS 0–3 after EVT^32^. Second, more patients with large artery atherosclerotic thrombosis were included in our study, which predicts a lower chance of successful reperfusion and a high number of thrombectomy passes^34,35^. In addition, these patients usually need to be treated with antithrombotic therapy. These may increase the risk of intracerebral hemorrhage. Third, despite the proportion of IVT (24.9%) in our study was comparable with previous trials (20.8–28.7%), it is also an important predictor of SICH.

### Limitations

The strengths of our study included the large-scale, prospective, multicenter design. This study also has several limitations. First, it has all the inherent limitations of a nonrandomized study. PSM or multivariable analyses can never adjust completely for systematic differences between treatment groups. Second, only Chinese patients were included, which may limit the generalizability.

## Conclusions

In patients with large cores on NCCT, EVT resulted in reasonable rates of favorable functional outcomes despite of higher risk of symptomatic intracerebral hemorrhage. Future clinical trials aimed at addressing the efficacy and safety of EVT in patients with large cores based on NCCT are warranted and under way.

## Ethical approval

The MAGIC study was approved by the ethics committee of the Xinqiao Hospital of the Army Medical University (ChiCTR2100051664) and the research board at each participating center approved the study protocol. Written informed consent was obtained from all the patients or their legal representatives.

## Consent

Written informed consent was obtained from all the patients or their legal representatives.

## Sources of funding

This study was funded by National Natural Science Foundation of China (No. 82271349), Academic Excellence Program (2022XKRC003) and Talent Incubation Program (2022YQB011).

## Author contribution

C.G., L.L., J.H., F.L., Q.Y., and W.Z.: conceived and designed the experiments; J.H., C.G., J.Y., J.S., Z.P., N.Y., C.L., L.L., W.K., J.H., L.C., M.G., J.H., C.Y., D.Y., X.L., J.M., M.W., X.L., Z.T., and X.B.: data collection; C.G. and D.W.: statistical analysis.

Wenjie Zi, Qingwu Yang, and Fengli Li acts as a guarantor and accepts full responsibility for the finished work and/or the conduct of the study, had access to the data, and controlled the decision to publish.

## Conflicts of interest disclosure

All authors declare that they have no conflict of interest.

## Research registration unique identifying number (UIN)

Chinese Clinical Trial Registry.

ChiCTR2100051664.

## Guarantor

Wenjie Zi, Fengli Li, and Qingwu Yang.

## Data available statement

Data are available on reasonable request.

## Provenance and peer review

Not commissioned, externally peer-reviewed.

## Assistance with the study

Not applicable.

## Presentation

Not applicable.

## Supplementary Material

## References

[1] Sarraj A Hassan A Savitz S . Outcomes of endovascular thrombectomy vs medical management alone in patients with large ischemic cores: a secondary analysis of the optimizing patient’s selection for endovascular treatment in acute ischemic stroke (SELECT) study. JAMA Neurol 2019;76:1147–1156. 31355873 10.1001/jamaneurol.2019.2109 PMC6664381

[2] Powers WJ Rabinstein AA Ackerson T . Guidelines for the early management of patients with acute ischemic stroke: 2019 update to the 2018 guidelines for the early management of acute ischemic stroke: a guideline for healthcare professionals from the American Heart Association/American Stroke Association. Stroke 2019;50:e344–e418. 31662037 10.1161/STR.0000000000000211

[3] Turc G Bhogal P Fischer U . European Stroke Organisation (ESO)- European Society for minimally invasive neurological therapy (ESMINT) guidelines on mechanical thrombectomy in acute ischemic stroke. J Neurointervent Surg 2019;11:535–538. 10.1136/neurintsurg-2018-014568 31152058

[4] Broocks G Hanning U Bechstein M . Association of thrombectomy with functional outcome for patients with ischemic stroke who presented in the extended time window with extensive signs of infarction. JAMA Netw Open 2022;5:e2235733. 36239941 10.1001/jamanetworkopen.2022.35733 PMC9568804

[5] Yoshimura S Sakai N Yamagami H . Endovascular therapy for acute stroke with a large ischemic region. N Engl J Med 2022;386:1303–1313. 35138767 10.1056/NEJMoa2118191

[6] Sarraj A Hassan AE Abraham MG . Trial of endovascular thrombectomy for large ischemic strokes. N Engl J Med 2023;388:1259–1271. 36762865 10.1056/NEJMoa2214403

[7] Huo X Ma G Tong X . Trial of endovascular therapy for acute ischemic stroke with large infarct. N Engl J Med 2023;388:1272–1283. 36762852 10.1056/NEJMoa2213379

[8] Bendszus M Fiehler J Subtil F . Endovascular thrombectomy for acute ischaemic stroke with established large infarct: multicentre, open-label, randomised trial. Lancet 2023;402:1753–1763. 37837989 10.1016/S0140-6736(23)02032-9

[9] Zaidat OO Kasab SA Sheth S . TESLA Trial: Rationale, Protocol, and Design. Stroke 2023;3:e000787. 10.1161/SVIN.122.000787 PMC12778678 41583679

[10] Almallouhi E Al Kasab S Hubbard Z . Outcomes of mechanical thrombectomy for patients with stroke presenting with low alberta stroke program early computed tomography score in the early and extended window. JAMA Netw Open 2021;4:e2137708. 34878550 10.1001/jamanetworkopen.2021.37708 PMC8655598

[11] Garcia-Esperon C Bivard A Johns H . Association of endovascular thrombectomy with functional outcome in patients with acute stroke with a large ischemic core. Neurology 2022;99:e1345–e1355. 35803723 10.1212/WNL.0000000000200908

[12] Rebello LC Bouslama M Haussen DC . Endovascular treatment for patients with acute stroke who have a large ischemic core and large mismatch imaging profile. JAMA Neurol 2017;74:34–40. 27820620 10.1001/jamaneurol.2016.3954

[13] Garcia-Tornel A Campos D Rubiera M . Ischemic core overestimation on computed tomography perfusion. Stroke 2021;52:1751–1760. 33682453 10.1161/STROKEAHA.120.031800

[14] Wintermark M Luby M Bornstein NM . International survey of acute stroke imaging used to make revascularization treatment decisions. Int J Stroke 2015;10:759–762. 25833105 10.1111/ijs.12491 PMC5286907

[15] Nguyen TN Abdalkader M Nagel S . Noncontrast computed tomography vs computed tomography perfusion or magnetic resonance imaging selection in late presentation of stroke with large-vessel occlusion. JAMA Neurol 2022;79:22–31. 34747975 10.1001/jamaneurol.2021.4082 PMC8576630

[16] Miao J Sang H Li F . Effect of imaging selection paradigms on endovascular thrombectomy outcomes in patients with acute ischemic stroke. Stroke 2023;54:1569–1577. 37165864 10.1161/STROKEAHA.122.042203

[17] Liu L Chen W Zhou H . Chinese stroke association guidelines for clinical management of cerebrovascular disorders: executive summary and 2019 update of clinical management of ischaemic cerebrovascular diseases. Stroke Vasc Neurol 2020;5:159–176. 32561535 10.1136/svn-2020-000378 PMC7337371

[18] Brott T Adams H Olinger C . Measurements of acute cerebral infarction: a clinical examination scale. Stroke 1989;20:864–870. 2749846 10.1161/01.str.20.7.864

[19] Higashida RT Furlan AJ Roberts H . Trial design and reporting standards for intra-arterial cerebral thrombolysis for acute ischemic stroke. Stroke 2003;34:e109–e137. 12869717 10.1161/01.STR.0000082721.62796.09

[20] Adams H Bendixen B Kappelle L . Classification of subtype of acute ischemic stroke. Definitions for use in a multicenter clinical trial. TOAST. Trial of Org 10172 in Acute Stroke Treatment. Stroke 1993;24:35–41. 7678184 10.1161/01.str.24.1.35

[21] Zaidat O Yoo A Khatri P . Recommendations on angiographic revascularization grading standards for acute ischemic stroke: a consensus statement. Stroke 2013;44:2650–2663. 23920012 10.1161/STROKEAHA.113.001972 PMC4160883

[22] von Kummer R Broderick J Campbell B . The Heidelberg Bleeding classification: classification of bleeding events after ischemic stroke and reperfusion therapy. Stroke 2015;46:2981–2986. 26330447 10.1161/STROKEAHA.115.010049

[23] Churilov L Arnup S Johns H . An improved method for simple, assumption-free ordinal analysis of the modified Rankin Scale using generalized odds ratios. Int J Stroke 2014;9:999–1005. 25196780 10.1111/ijs.12364

[24] Wang D Pocock S . A win ratio approach to comparing continuous non-normal outcomes in clinical trials. Pharm Stat 2016;15:238–245. 26970432 10.1002/pst.1743

[25] Funk MJ Westreich D Wiesen C . Doubly robust estimation of causal effects. Am J Epidemiol 2011;173:761–767. 21385832 10.1093/aje/kwq439 PMC3070495

[26] Mathew G Agha R Albrecht J . STROCSS 2021: strengthening the reporting of cohort, cross-sectional and case-control studies in surgery. International journal of surgery (London, England) 2021;96:106165. 34774726 10.1016/j.ijsu.2021.106165

[27] Nogueira RG Haussen DC Liebeskind D . Stroke imaging selection modality and endovascular therapy outcomes in the early and extended time windows. Stroke 2021;52:491–497. 33430634 10.1161/STROKEAHA.120.031685

[28] Jadhav AP Goyal M Ospel J . Thrombectomy with and without computed tomography perfusion imaging in the early time window: a pooled analysis of patient-level data. Stroke 2022;53:1348–1353. 34844423 10.1161/STROKEAHA.121.034331

[29] Dhillon PS Butt W Podlasek A . Perfusion imaging for endovascular thrombectomy in acute ischemic stroke is associated with improved functional outcomes in the early and late time windows. Stroke 2022;53:2770–2778. 35506384 10.1161/STROKEAHA.121.038010 PMC9389941

[30] Nezu T Koga M Nakagawara J . Early ischemic change on CT versus diffusion-weighted imaging for patients with stroke receiving intravenous recombinant tissue-type plasminogen activator therapy: stroke acute management with urgent risk-factor assessment and improvement (SAMURAI) rt-PA registry. Stroke 2011;42:2196–2200. 21719764 10.1161/STROKEAHA.111.614404

[31] Goyal M Menon BK Coutts SB . Effect of baseline CT scan appearance and time to recanalization on clinical outcomes in endovascular thrombectomy of acute ischemic strokes. Stroke 2011;42:93–97. 21088240 10.1161/STROKEAHA.110.594481

[32] Uchida K Shindo S Yoshimura S . Association between alberta stroke program early computed tomography score and efficacy and safety outcomes with endovascular therapy in patients with stroke from large-vessel occlusion: a secondary analysis of the recovery by endovascular salvage for cerebral ultra-acute embolism-Japan large ischemic core trial (RESCUE-Japan LIMIT). JAMA Neurol 2022;79:1260–1266. 36215044 10.1001/jamaneurol.2022.3285 PMC9552045

[33] Kaesmacher J Chaloulos-Iakovidis P Panos L . Mechanical thrombectomy in ischemic stroke patients with alberta stroke program early computed tomography score 0-5. Stroke 2019;50:880–888. 30827193 10.1161/STROKEAHA.118.023465 PMC6430594

[34] Lee JS Lee SJ Hong JM . Endovascular treatment of large vessel occlusion strokes due to intracranial atherosclerotic disease. J Stroke 2022;24:3–20. 35135056 10.5853/jos.2021.01375 PMC8829471

[35] de Havenon A Zaidat OO Amin-Hanjani S . Large vessel occlusion stroke due to intracranial atherosclerotic disease: identification, medical and interventional treatment, and outcomes. Stroke 2023;54:1695–1705. 36938708 10.1161/STROKEAHA.122.040008 PMC10202848

