# Supplementary material for: Endovascular treatment versus standard medical treatment in patients with established large infarct: a cohort study
Source: Int J Surg. 2024 May 8;110(8):4775–84. doi: 10.1097/JS9.0000000000001539 (PMC11326037; doi:10.1097/JS9.0000000000001539)
Supplement: Supplementary file 2 [file js9-110-4775-s002.docx]

**Power Analysis**

The power and sample size were determined based on the previous SELECT2 study. In this study, the rate of achieving a favorable outcome was 0.376 in the EVT group and 0.184 in the SMT group. The Figure illustrates how the power of the study changes with different total sample sizes, maintaining a ratio of EVT:SMT = 2:1 and using a two-sided alpha level of 0.05.

Based on these calculations, it was found that having 490 patients in the EVT group and 255 patients in the SMT group would provide a power greater than 0.999 at a two-sided alpha level of 0.05.
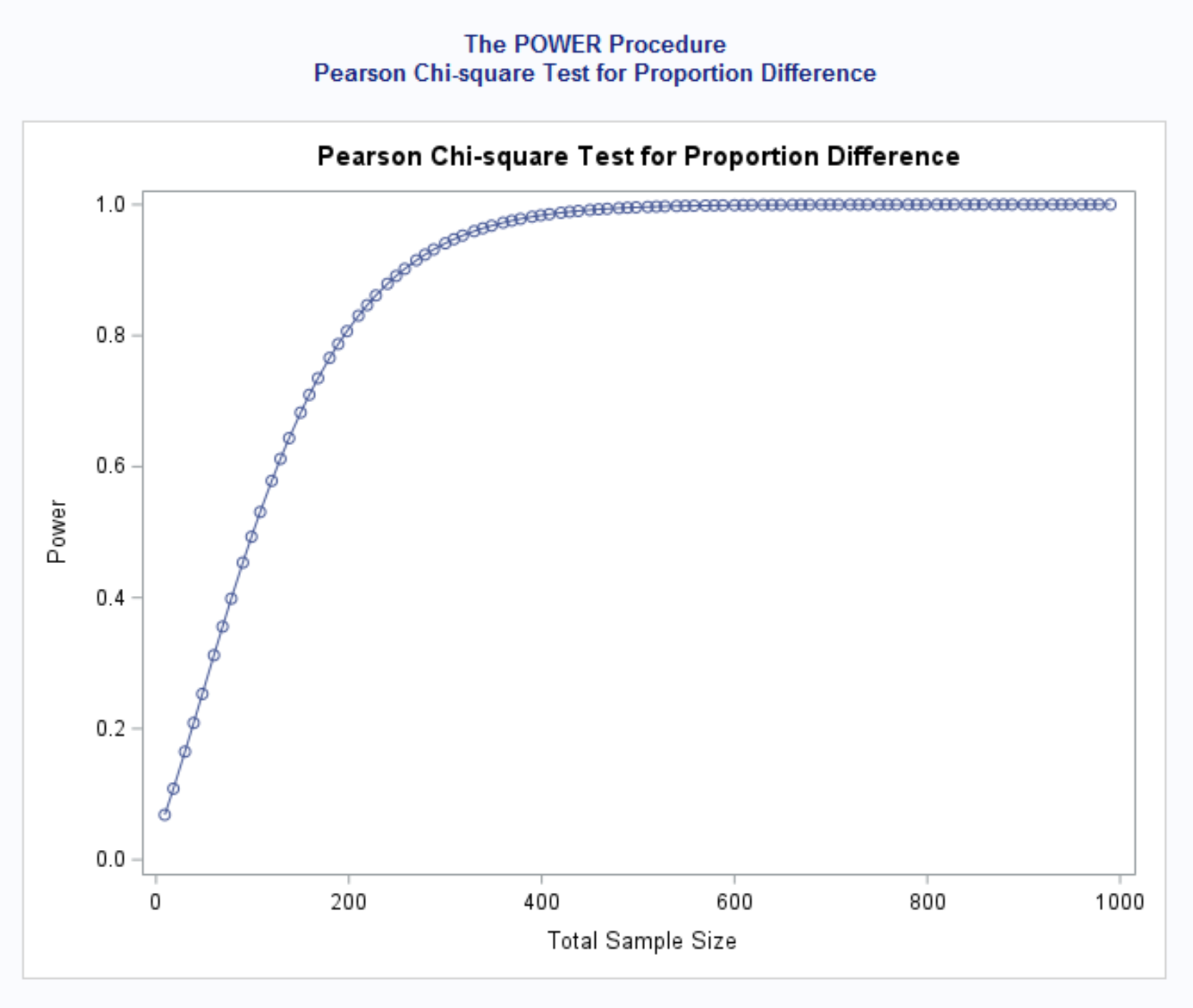


Figure S1. The Power changes with the sample size

**Instrumental Variable Analysis**

To support causal inference by accounting for observed and unobserved confounding factors, the IV model was used in the sensitivity analysis to assess the association between Endovascular Treatment (EVT) use and clinical outcomes. IV analysis is an increasingly popular technique in medical research that overcomes confounding issues in traditional observational studies by taking advantage of naturally occurring variations in treatment patterns that approximate randomization more closely.^1^ The heterogeneity of the clinical pattern of EVT has resulted in a "marginal population" who would be more likely to receive an EVT when treated at a hospital with the high preference for EVT than treated with EVT in a hospital with lower preference. When certain assumptions were met, IV categorized patients into separate treatment groups regardless of their individual characteristics. This means that outcomes were evaluated based on the likelihood of receiving treatment rather than actual treatments

received.^2, 3^

In this study, site-level preference for EVT use served as our instrument.7^1, 4^ Our 2-stage approach involved estimating a conditional expectation of treatment allocation based on co-variates and the instrumental variable, followed by predicting outcomes using original treatment allocation, covariates, and residuals from the first-stage regression.^5^ To assess instrument strength/exogeneity (correlation), we utilized the Wald F-test. An instrument was classified as strong if its F-statistic exceeded 10. ^6^

At the second stage, different models were chosen based on the distribution of outcomes. For comparing dichotomous outcomes, a generalized linear model with robust sandwich variance was used. The Poisson distribution and log link function were employed to estimate relative risk, while the Gaussian distribution and identity function were used for estimating risk difference. For comparing distributions of mRS scores at 90 days, ordered logistic regression was employed to estimate the common odds ratio. We will use bootstrap method with 3000 iterations and a seed value of 10101 to obtain coefficient estimates. Additionally, asymptotically correct standard errors were calculated for obtaining coefficient estimates.^5^

**Propensity Score Matching**

Propensity score matching (PSM) methods was used to assess the effectiveness and safety outcomes of patients treated with EVT versus those treated with SMT. We estimated the propensity score using a multivariable logistic regression model that included variables with potential unbalanced variables with a p-value < 0.1, with treatment received (SMT or EVT) as the dependent variable. Patients who received SMT were matched 1:1 to those who received EVT based on their propensity scores without replacement, using greedy nearest neighbor matching with a 0.2 caliper. The Figure below shows the standard mean deviation before and after matching.


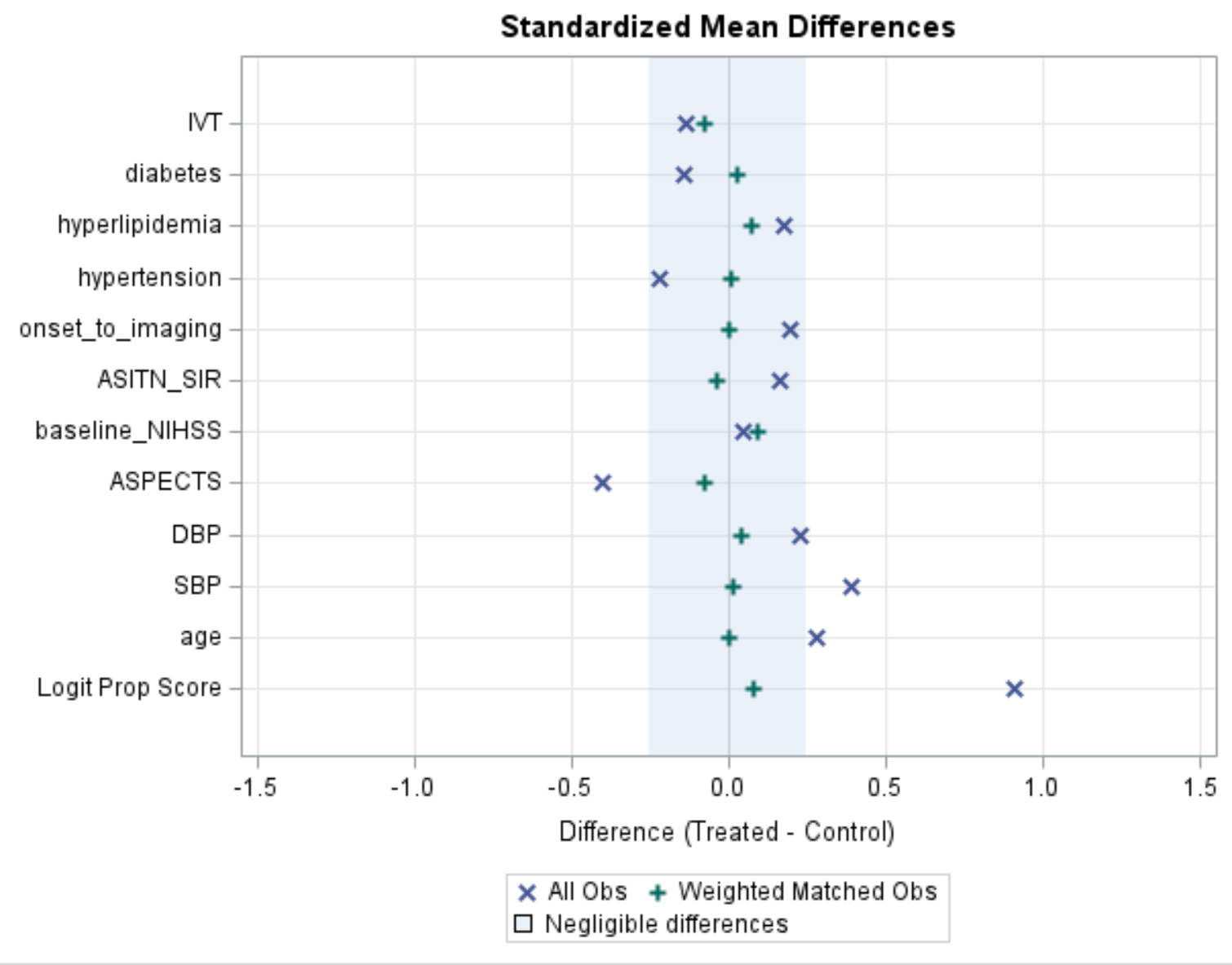


Figure S2. Propensity matching score analysis

**Inverse Probability Weighting**

The treatment effect was estimated in the IPTW cohort using the inversed probability-weighted regression adjustment model. This model utilized the inversed propensity score to weight each subject and adjusted for weighted regression coefficients to compute averages of predicted outcomes at the treatment level. Relative risk, risk differences, common odds ratio with 95% CIs were derived based on predicted event rates or means using patients treated with SMT as a reference group.


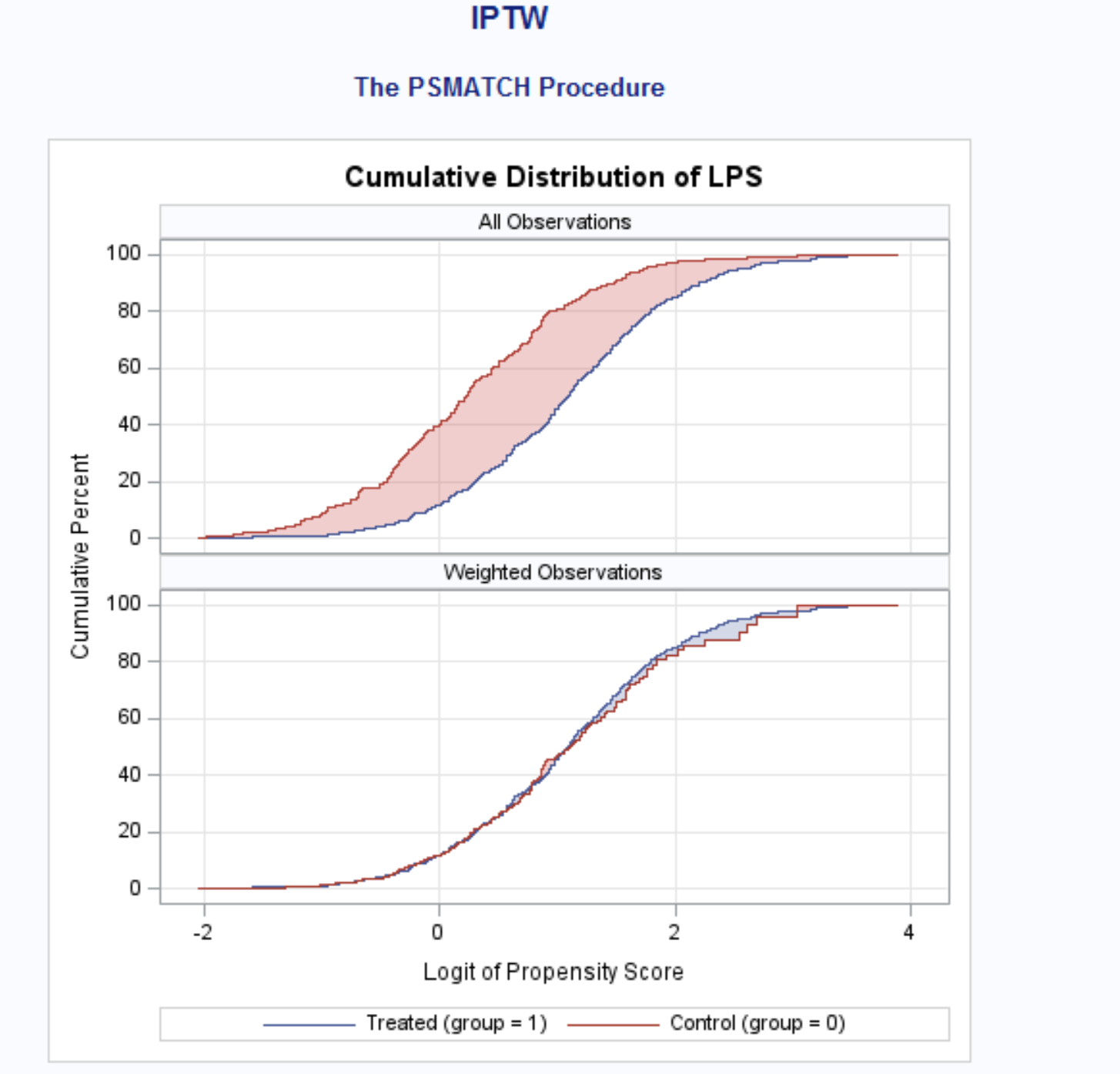


Figure S3. The cumulative distribution of logit propensity score before and after matching


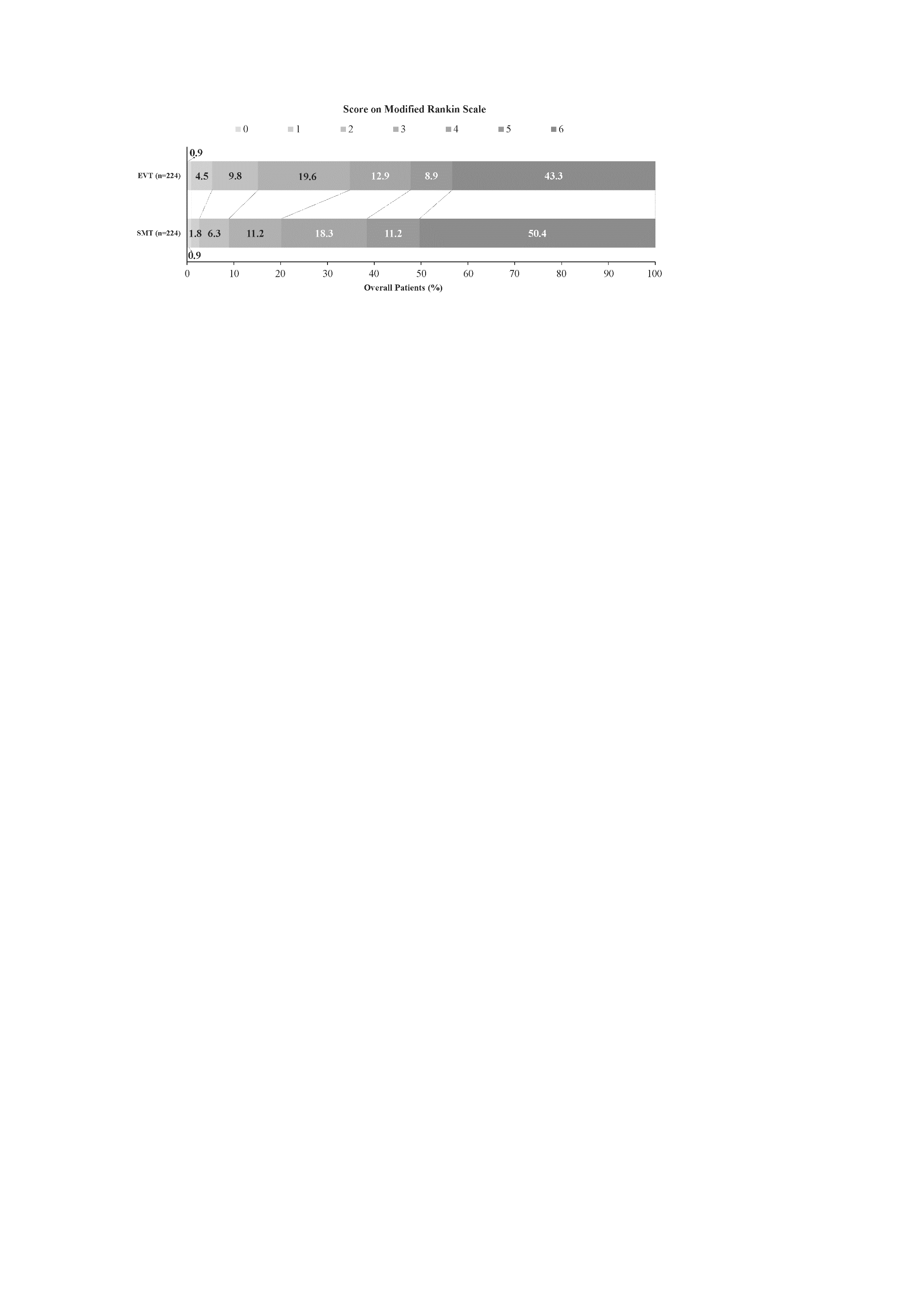


Figure S4. Distribution of the Modified Rankin Scale score at 90 days in the propensity score matching data set

Table S1. Baseline characteristics of the patients based on propensity score matching data set.

| **Characteristics** | **All (n = 448)** | **EVT (n = 224)** | **SMT (n = 224)** | ***P* value** |
| --- | --- | --- | --- | --- |
| Age, median (IQR), y | 71 (64–79) | 71 (64–80) | 72 (65–79) | 0.87 |
| Sex |  |  |  | 0.34 |
| Male | 244 (54.5) | 127 (56.7) | 117 (52.2) |  |
| Female | 204 (45.5) | 97 (43.3) | 107 (47.8) |  |
| Medical History |  |  |  |  |
| Atrial fibrillation | 189 (42.2) | 90 (40.2) | 99 (44.2) | 0.39 |
| Hypertension | 311 (69.4) | 156 (69.6) | 155 (69.2) | 0.92 |
| Hyperlipidemia | 76 (17.0) | 41 (18.3) | 35 (15.6) | 0.45 |
| Diabetes | 82 (18.3) | 42 (18.8) | 40 (17.9) | 0.81 |
| Smoking | 128 (28.6) | 67 (29.9) | 61 (27.2) | 0.53 |
| Blood pressure on admission, median (IQR), mm Hg * |  |  |  |  |
| Systolic | 152 (136–172) | 151 (137–170) | 153 (135–175) | 0.92 |
| Diastolic | 88 (78–100) | 88 (78–99) | 88 (80–101) | 0.55 |
| Glucose, median (IQR), mmol/L † | 7.1 (6.0–8.8) | 7.3 (6.1–9.1) | 7.1 (5.9–8.4) | 0.20 |
| Baseline NIHSS score, median (IQR) | 17 (14–21) | 17 (14–20) | 17 (14–22) | 0.60 |
| Baseline ASPECTS, median (IQR) | 3 (1–5) | 3 (2–5) | 3 (1–5) | 0.54 |
| 0–2 |  |  |  |  |
| 3–5 |  |  |  |  |
| Left hemisphere affected | 201 (44.9) | 102 (45.5) | 99 (44.2) | 0.78 |
| Intravenous thrombolysis | 132 (29.5) | 62 (27.7) | 70 (31.3) | 0.41 |
| ASTIN/SIR grade ‡, median (IQR) | 2.0 (1.0, 3.0) | 2.0 (1.0, 3.0) | 2.0 (1.0, 3.0) | 0.77 |
| 0–1 | 192 (42.9) | 95 (42.4) | 97 (43.3) |  |
| 2 | 137 (30.6) | 72 (32.1) | 65 (29.0) |  |
| 3–4 | 119 (26.6) | 57 (25.4) | 62 (27.7) |  |
| Stroke causative mechanism |  |  |  | 0.83 |
| Large artery atherosclerosis | 206 (46.0) | 107 (47.8) | 99 (44.2) |  |
| Cardioembolism | 200 (44.6) | 98 (43.8) | 102 (45.5) |  |
| Other | 10 (2.2) | 5 (2.2) | 5 (2.2) |  |
| Unknown | 32 (7.1) | 14 (6.3) | 18 (8.0) |  |
| Occlusion location |  |  |  | 0.72 |
| Internal carotid artery | 132 (29.5) | 68 (30.4) | 64 (28.6) |  |
| M1 segment | 262 (58.5) | 127 (56.7) | 135 (60.3) |  |
| M2 segment | 42 (12.1) | 29 (12.9) | 25 (11.2) |  |
| Tandem occlusions | 33 (7.4) | 17 (7.6) | 16 (7.1) | 0.86 |
| General anesthesia | — | 35 (15.6) | — |  |
| Last seen well to imaging time, median (IQR), min § | 310 (170–509) | 335 (178–505) | 303 (161–513) | 0.55 |
| Last seen well to puncture time, median (IQR), min ¶ | — | 399 (261–605) | — |  |
| Last seen well to recanalization time, median (IQR), min ‖ | — | 499 (356–713) | — |  |

Table S2 Details of Propensity Score Matching Analysis

| **Outcomes** | **Treatment Effect** | **All** | **EVT+SMT** | **SMT** | **Effect Value** | ***P* Value** |
| --- | --- | --- | --- | --- | --- | --- |
|  |  | **(n = 448)** | **(n = 224)** | **(n = 224)** |  |  |
| **Primary Outcome** |  |  |  |  |  |  |
| Modified Rankin scale score of 0–3 at 90 days | Risk Ratio | 123 (27.5) | 78 (34.8) | 45 (20.1) | 1.79 (1.35 to 2.37) | <0.001 |
|  |  |  |  |  |  |  |
|  | Risk Difference |  |  |  | 13.64 (6.36 to 20.93) | <0.001 |
| **Secondary Outcome** |  |  |  |  |  |  |
| Score on the modified Rankin scale at 90 days (IQR) | Common Odds Ratio | 5.0 (3.0, 6.0) | 5.0 (3.0, 6.0) | 6.0 (4.0, 6.0) | 1.74 (1.20 to 2.51) | 0.004 |
|  |  |  |  |  |  |  |
|  | Generalized Odds Ratio |  |  |  | 1.29 (1.06 to 1.59) | 0.01 |
|  | Win Ratio |  |  |  | 1.43 (1.08 to 1.92) | 0.01 |
| Modified Rankin scale score of 0–2 at 90 days | Risk Ratio | 54 (12.1) | 34 (15.2) | 20 (8.9) | 1.90 (1.16 to 3.13) | 0.01 |
|  |  |  |  |  |  |  |
|  | Risk Difference |  |  |  | 5.86 (0.23 to 11.49) | 0.04 |
| Modified Rankin scale score of 0–4 at 90 days | Risk Ratio | 193 (43.1) | 107 (47.8) | 86 (38.4) | 1.25 (1.03 to 1.50) | 0.02 |
|  |  |  |  |  |  |  |
|  | Risk Difference |  |  |  | 7.93 (0.17 to 15.69) | 0.045 |
| Successful reperfusion |  | — | 187 (83.5) | — |  |  |
| **Safety Outcome** |  |  |  |  |  |  |
| Symptomatic intracranial hemorrhage within 48 hours — no. (%) | Risk Ratio | 31 (6.9) | 25 (11.2) | 6 (2.7) | 4.33 (1.78 to 10.55) | <0.001 |
|  |  |  |  |  |  |  |
|  | Risk Difference |  |  |  | 8.61 (4.00 to 13.23) | <0.001 |
| Death within 90 days — no. (%) | Risk Ratio | 210 (46.9) | 97 (43.3) | 113 (50.4) | 0.92 (0.76 to 1.10) | 0.35 |
|  |  |  |  |  |  |  |
|  | Risk Difference |  |  |  | -5.33 (-13.29 to 2.62) | 0.19 |
| Any intracranial hemorrhage within 48 hours — no. (%) | Risk Ratio | 107 (23.9) | 82 (36.6) | 25 (11.2) | 3.37 (2.26 to 5.02) | <0.001 |

Table S3. Additional Safety Outcomes.

| **Outcome** | **All** | **EVT** | **SMT** | **Risk Ratio** | **P Value** |
| --- | --- | --- | --- | --- | --- |
| Herniation during hospitalization — no. (%) | 185 (24.8) | 140 (28.6) | 45 (17.6) | 1.62 (1.20 - 2.19) | 0.002 |
| Decompressive hemicraniectomy during hospitalization — no. (%) | 59 (7.9) | 48 (9.8) | 11 (4.3) | 2.27 (1.20 - 4.30) | 0.01 |

Table S4. Two-Stage Residual Inclusion Instrumental Variable Analysis.

| **Outcome** | **Treatment Effect** | **Effect Value**  **(Bootstrap Method)** | **P Value** | **Effect Value**  **(ACSE)** | **P**  **Value** |
| --- | --- | --- | --- | --- | --- |
| **Primary Outcome** |  |  |  |  |  |
| Modified Rankin scale score of 0–3 at 90 days — no. (%) | Risk Ratio | 2.31 (1.40 to 3.82) | <0.001 | 2.31 (1.42 to 3.76） | <0.001 |
|  | Risk Difference | 20.17 (7.19 to 33.14) | <0.001 | 20.17 (7.64 to 32.69) | 0.002 |
| **Secondary Outcome** |  |  |  |  |  |
| Score on the modified Rankin scale at 90 days (IQR) |  |  |  |  |  |
|  | Common Odds Ratio | 3.35 (0.61 to 6.82) | <0.001 | - | - |
| Modified Rankin scale score of 0–2 at 90 days — no. (%) | Risk Ratio | 3.77 (1.70 to 8.41) | <0.001 | 3.78 (1.79 to 7.97) | <0.001 |
|  | Risk Difference | 18.02 (7.22 to 28.82) | <0.001 | 18.02 (7.79 to 28.25) | <0.001 |
| Modified Rankin scale score of 0–4 at 90 days — no. (%) | Risk Ratio | 1.78 (1.25 to 2.55) | <0.001 | 1.78 (1.26 to 2.52) | <0.001 |
|  | Risk Difference | 23.07 (8.93 to 37.22) | <0.001 | 23.07 (9.23 to 36.87) | <0.001 |
| Successful reperfusion — no. (%) |  |  |  |  |  |
| **Safety Outcome** |  |  |  |  |  |
| Symptomatic intracranial hemorrhage within 48 hours — no. (%) | Risk Ratio | 2.57 (0.39 to 16.6) | 0.33 | 2.54 (0.82 to 7.89) | 0.11 |
|  | Risk Difference | 2.62 (-6.83 to 12.07) | 0.59 | 2.62 (-6.46 to 11.88) | 0.58 |
| Death within 90 days — no. (%) | Risk Ratio | 0.71 (0.51 to 0.98) | 0.038 | 0.71 (0.51 to 0.97) | 0.03 |
|  | Risk Difference | -18.27 (-33.08 to -3.46) | 0.016 | -18.27 (-32.76 to -3.78) | 0.01 |
| Any intracranial hemorrhage within 48 hours — no. (%) | Risk Ratio | 1.77 (0.99 to 3.16) | 0.055 | 1.77 (1.02 to 3.07) | 0.04 |
|  | Risk Difference | 6.88 (-7.51 to 21.27) | 0.349 | 6.88 (-6.98 to 20.74) | 033 |

Abbreviation: ACSE, Asymptotically correct standard error.

Table S5. Efficacy and safety outcomes (Generalized Estimating Equation).

| **Outcome** | **Treatment Effect** | **Effect Value** | **P Value** |
| --- | --- | --- | --- |
| **Primary Outcome** |  |  |  |
| Modified Rankin scale score of 0–3 at 90 days — no. (%) | Risk Ratio | 1.85 (1.56–2.20) | <0.001 |
|  | Risk Difference | 13.30 (7.81–18.80) | <0.001 |
| **Secondary Outcome** |  |  |  |
| Score on the modified Rankin scale at 90 days (IQR) | Generalized Odds Ratio |  |  |
|  | Common Odds Ratio | 1.76 (1.42–2.20) | <0.001 |
| Modified Rankin scale score of 0–2 at 90 days — no. (%) | Risk Ratio | 2.38 (1.73–3.28) | <0.001 |
|  | Risk Difference | 10.29 (6.00–14.59) | <0.001 |
| Modified Rankin scale score of 0–4 at 90 days — no. (%) | Risk Ratio | 1.28 (1.13–1.45) | <0.001 |
|  | Risk Difference | 8.16 (3.62–12.69) | <0.001 |
| Successful reperfusion — no. (%) |  |  |  |
| **Safety Outcome** |  |  |  |
| Symptomatic intracranial hemorrhage within 48 hours — no. (%) | Risk Ratio | 5.44 (2.12–13.94) | <0.001 |
|  | Risk Difference | 10.29 (6.47–14.12) | <0.001 |
| Death within 90 days — no. (%) | Risk Ratio | 0.95 (0.80–1.12) | 0.53 |
|  | Risk Difference | -5.02 (-11.86–1.82) | 0.15 |
| Any intracranial hemorrhage within 48 hours — no. (%) | Risk Ratio | 3.46 (2.36–5.08) | <0.001 |
|  | Risk Difference | 26.93 (20.69–33.17) | <0.001 |
|  | Risk Difference | 3.00 (-1.84–7.84) | 0.23 |

Table S6. Generalized Linear Mixed Effect Model.

| **Outcome** | **Treatment Effect** | **Effect Value** | **P Value** |
| --- | --- | --- | --- |
| **Primary Outcome** |  |  |  |
| Modified Rankin scale score of 0–3 at 90 days — no. (%) | Risk Ratio | 1.79 (1.51–2.11) | <0.001 |
|  | Risk Difference | 12.83 (6.30–19.35) | <0.001 |
| **Secondary Outcome** |  |  |  |
| Score on the modified Rankin scale at 90 days (IQR) |  |  |  |
|  | Common Odds Ratio | 1.67 (1.15–2.38) | 0.007 |
| Modified Rankin scale score of 0–2 at 90 days — no. (%) | Risk Ratio | 1.90 (1.28–2.83) | 0.002 |
|  | Risk Difference | - | - |
| Modified Rankin scale score of 0–4 at 90 days — no. (%) | Risk Ratio | 1.25 (1.10–1.41) | 0.001 |
|  | Risk Difference | 7.44 (0.27–14.61) | 0.04 |
| Successful reperfusion — no. (%) |  |  |  |
| **Safety Outcome** |  |  |  |
| Symptomatic intracranial hemorrhage within 48 hours — no. (%) | Risk Ratio | 4.33 (1.68–11.14) | 0.002 |
|  | Risk Difference | 8.61 (4.59–12.63) | <0.001 |
| Death within 90 days — no. (%) | Risk Ratio | 0.92 (0.79–1.07) | 0.27 |
|  | Risk Difference | -4.45 (-12.62–3.72) | 0.29 |
| Any intracranial hemorrhage within 48 hours — no. (%) | Risk Ratio | 3.37 (2.32–4.91) | <0.001 |
|  | Risk Difference | 27.24 (20.07–34.41) | <0.001 |

**Table S7.** Procedure Details of the Endovascular Treatment.

| Characteristics | EVT+SMT  (N = 490) |
| --- | --- |
| Type of Endovascular Treatment |  |
| Stent retriever | 87 (17.8%) |
| Balloon/ Stenting | 28 (5.7%) |
| Intra-arterial medication or Mechanical fragmentation | 10 (2.0%) |
| Aspiration | 251 (51.2%) |
| Combination of Stent-Retriever and Aspiration | 109 (22.2%) |
| Spontaneous reperfusion | 1 (0.2%) |
| Missing | 4 (1%) |

**Table S8.** Primary Efficacy Outcome (Modified Rankin Scale Score of 0 to 3) Stratified by Different Endovascular Modality.

| **Modified Rankin Scale score of 0 to 3 at 90 days** | **Yes**  **(N = 181)** | **No**  **(N = 309)** |
| --- | --- | --- |
| Stent retriever | 33 (37.93) | 54 (62.07) |
| Balloon/Stenting | 10 (35.71) | 18 (64.29) |
| Intra-arterial medication or Mechanical fragmentation | 3 (30.0) | 7 (70) |
| Aspiration | 92 (36.65) | 159 (63.35) |
| Combination of Stent-Retriever and Aspiration | 41 (37.61) | 68 (62.39) |
| Spontaneous reperfusion | 0 (0) | 1 (100.0) |
| Missing | 2 (50.0) | 2 (50.0) |

**Table S9.** Symptomatical Intracranial Hemorrhage Stratified by Different Endovascular Modality.

| **SICH** | **Yes**  **(N = 65)** | **No**  **(N = 425)** |
| --- | --- | --- |
| Stent retriever | 12 (13.80) | 75 (86.21) |
| Balloon/ Stenting | 2 (7.14) | 26 (92.86) |
| Intra-arterial medication or Mechanical fragmentation | 1 (10.00) | 9 (90) |
| Aspiration | 34 (13.55) | 217 (86.45) |
| Combination of Stent-Retriever and Aspiration | 16 (14.68) | 93 (85.32) |
| Spontaneous reperfusion | 0 (0) | 1 (100) |
| Missing | 0 (0) | 4 (100) |

**Table S10.** Symptomatical Intracranial Hemorrhage Stratified by Different Endovascular Modality.

| **Death at 90 days** | **Yes**  **(N = 205)** | **No**  **(N = 285)** |
| --- | --- | --- |
| Stent retriever | 29 (33.33) | 58 (66.67) |
| Balloon/Stenting | 11 (39.29) | 17 (60.71) |
| Intra-arterial medication or Mechanical fragmentation | 2 (20.0) | 8 (80.0) |
| Aspiration | 117 (46.61) | 134 (53.39) |
| Combination of Stent-Retriever and Aspiration | 43 (39.45) | 66 (60.55) |
| Spontaneous reperfusion | 1 (100.0) | 0 (0.0) |
| Missing | 2 (50.0) | 2 (50.0) |

**Table S11.** Logistic regression yielding the treatment effect of different endovascular modalities.

| Endovascular modalities | Unadjusted OR  (95%CI) | P-value | Adjusted OR  (95%CI) | P-value |
| --- | --- | --- | --- | --- |
| Stent retriever | Reference |  | Reference |  |
| Balloon/ Stenting | 0.91 (0.38 to 2.21) | 0.97 | 0.57 (0.20 to 1.64) | 0.98 |
| Intra-arterial medication or Mechanical fragmentation | 0.70 (0.17 to 2.90) | 0.97 | 0.81 (0.15 to 4.28) | 0.98 |
| Aspiration | 0.95 (0.57 to 1.57) | 0.97 | 1.15 (0.60 to 2.19) | 0.97 |
| Combination of Stent-Retriever and Aspiration | 0.99 (0.55 to 1.76) | 0.97 | 1.39 (0.67 to 2.88) | 0.97 |
| Spontaneous reperfusion | - |  | - |  |

**Reference**

1. Chen Y and Briesacher BA. Use of instrumental variable in prescription drug research with observational data: a systematic review. *J Clin Epidemiol*. 2011;64:687-700.

2. Maciejewski ML, Dowd BE and Norton EC. Instrumental Variables and Heterogeneous Treatment Effects. *JAMA*. 2022;327:1177-1178.

3. Maciejewski ML and Brookhart MA. Using Instrumental Variables to Address Bias From Unobserved Confounders. *JAMA*. 2019;321:2124-2125.

4. Butala NM, Makkar R, Secemsky EA, Gallup D, Marquis-Gravel G, Kosinski AS, Vemulapalli S, Valle JA, Bradley SM, Chakravarty T, Yeh RW and Cohen DJ. Cerebral Embolic Protection and Outcomes of Transcatheter Aortic Valve Replacement: Results From the Transcatheter Valve Therapy Registry. *Circulation*. 2021;143:2229-2240.

5. Terza JV. Two-Stage Residual Inclusion Estimation in Health Services Research and Health Economics. *Health Serv Res*. 2018;53:1890-1899.

6. Staiger D and Stock JHJSSEP. Instrumental Variables Regression with Weak Instruments.

7. Funk MJ, Westreich D, Wiesen C, Sturmer T, Brookhart MA and Davidian M. Doubly robust estimation of causal effects. *Am J Epidemiol*. 2011;173:761-7.
